# OP46 A New Modular Approach To Updating National Institute For Health And Care Excellence Manuals

**DOI:** 10.1017/S0266462324001089

**Published:** 2025-01-07

**Authors:** Lizzie Walker, Laura Flight, Carl Prescott, Koonal Shah

## Abstract

**Introduction:**

Methods and processes for health technology assessment (HTA) must be sufficiently flexible to reflect emerging best practice and adapt to changes in the health and care landscape. They must also be clearly documented and subject to review, assessment, and consultation. This requires a framework to flexibly update HTA manuals, while providing stability and predictability for stakeholders.

**Methods:**

The National Institute for Health and Care Excellence (NICE) is introducing a modular approach to updating its manuals. Previously, NICE completed full, but infrequent, updates to its manuals. A modular update is a review of the process and/or methods informing NICE’s guidance in a specific subject area, which can then be updated in the appropriate manual(s) if required. We are developing a modular updates framework that sets out NICE’s approach for identifying and prioritizing modular updates to review, reviewing the evidence and proposing changes to the manual(s), engaging and consulting with stakeholders, and implementing the modular updates.

**Results:**

The proposed approach allows external stakeholders to contribute to topic identification and the content of updates, helping to ensure that the manuals meet the needs of users. The first modular update to the NICE manual for health technology evaluations was published in October 2023. This included updated processes for carrying out cost comparisons, streamlining committee decision-making and handling confidential information. Potential future modular updates may include updated methods to consider health inequalities.

**Conclusions:**

The introduction of a modular approach will enable NICE to be more agile and responsive in monitoring, reviewing, and improving our methods and processes, making sure they remain cutting edge as the health and care landscape continues to evolve.

